# National Approach in Response to the COVID-19 Pandemic in Iran

**DOI:** 10.30476/IJCBNM.2020.85928.1308

**Published:** 2020-07

**Authors:** Tayebe Pourghaznein, Sina Salati

**Affiliations:** 1 Nursing and Midwifery Care Research Center, Mashhad University of Medical Sciences, Mashhad, Iran; 2 Student Research Committee, School of Nursing and Midwifery, Mashhad University of Medical Sciences, Mashhad, Iran

**DEAR EDITOR**

Since the outbreak of the New Corona Virus in Wuhan, China and its spreading to Iran, first cases of COVID-19 positive were identified in Qom, Iran and in a short period of time many other main provinces including Isfahan, Tehran, Markazi, and Semnan were also infected with the virus shortly after that in all 31 provinces the virus spread. ^1^ Super Spreading power of this disease is the reason why we have many infected people daily and according to global epidemiological information, the number of infected people will reach to 74877 in April 15,2020. ^2^ like many other countries, Iran started medical and hygienic measures with the establishment of a National committee for fighting Corona Virus. Our country imposed limited commuting to religious, recreational places and trading centers, closed schools and universities, decreased working hours, and cleaned crowded areas and passageways with disinfectants (subway stations, bus stations, and gas stations). Other efforts include: Establishing outposts in order to identify cases of Corona Virus at City entrances and Telephone screening through 4030"that is a hotline for COVID-19 detection and providing relevant health information". ^1^ Despite the necessary precautions taken, there were problems in fighting the Corona Disease, the most important of which include: Insufficiency regarding personal protective equipment like masks, disinfectants and hospital gowns, ^3^ Inadequate number of hospital beds and equipment in some of the cities and shortage of health workers. This issue has caused a high concern in the society and amongst the health workers who take care of Corona virus cases in hospitals. Also, due to the experienced mental disorders of the health care workers while COVID-19 outbreak, ^4^ the role of such shortages in the fight against this disease is crucial. In a situation like this where the number of infected people shoots up, hygienic needs of Iran increases, too. A new trend in fighting the disease has begun. In this trend, different groups of people like clerics and cleric students, Basij, medical and non-medical students have taken measures in fighting the Corona Virus which are in line with the World health organization and Ministry of Health and Medical Education standards. These measures are taken in 4 general areas, including: 1. disinfecting the pssageways (Streets, Alleys), highly populated areas (Trading Centers, Bus and Subway stations, Gas stations) and vehicles, 2. producing personal protective equipment (Masks, face shields, gowns and disinfectants), 3. helping with the Telephone Screening by Medical students, and 4. Volunteer medical students participation in health care centers and hospitals.

Now, despite the exigent circumstances regarding the spread of COVID-19 worldwide, volunteer measures and organized activities in Iran have led to provision of telephone screening information and details about the COVID-19 to more than 65 million people until March 31, 2020; ^5^ and production of thousands of hygienic products daily has had a significant role in controlling and fighting the Corona Virus Pandemic. Although in the early period of the virus outbreak, Iran was the third country to have the most cases of Corona positive cases after China and Korea until 2^nd^ of March, and the number of identified cases increased in April 15, 2020 which might be due to the efficient and comprehensive screening process, it became the 8^th^ country with the most positive cases of Corona after the United States, Spain, Italy, France, Germany, England, and China. ^2^ It is also expected that with adequate health equipment, specialized human resources and provision of scientific information to the community, the public and health workers who take care of Corona virus cases will have tranquility.

Therefore, Iran’s approach towards facing the COVID-19 clearly states the fact that national approach in fighting the Corona Virus is an important factor among other measures taken and it is mentioned as a modern approach in managing a health crisis in a large scale. 
